# Azacitidine in combination with shortened venetoclax treatment cycles in patients with acute myeloid leukemia

**DOI:** 10.1007/s00277-024-06048-5

**Published:** 2024-10-25

**Authors:** Maximilian Fleischmann, Madlen Jentzsch, Annamaria Brioli, Florian Eisele, Jochen J. Frietsch, Farina Eigendorff, Romy Tober, Karin G. Schrenk, Jakob Friedrich Hammersen, Olaposi Yomade, Inken Hilgendorf, Andreas Hochhaus, Sebastian Scholl, Ulf Schnetzke

**Affiliations:** 1 Klinik für Innere Medizin II, Abteilung für Hämatologie Und Internistische Onkologie, Universitätsklinikum Jena, Jena, Germany; 2 Comprehensive Cancer Center Central Germany, Campus Jena, Jena, Germany; 3 Klinik Und Poliklinik für Hämatologie, Zelltherapie, Hämostaseologie Und Infektiologie, Universitätsklinikum Leipzig, Leipzig, Germany; 4 Comprehensive Cancer Center Central Germany, Campus Leipzig, Leipzig, Germany; 5 Klinik Und Poliklinik für Innere Medizin C, Abteilung für Hämatologie Und Onkologie, Universitätsklinikum Greifswald, Greifswald, Germany; 6 Medizinische Klinik Und Poliklinik II, Universitätsklinikum Würzburg, Würzburg, Germany

**Keywords:** Acute myeloid leukemia, Venetoclax, 5-azacitidine, 7 + 7, Toxicity, Shortened treatment

## Abstract

The combination of venetoclax with hypomethylating agents is currently the standard of care for elderly patients with acute myeloid leukemia (AML) ineligible for intensive chemotherapy. Despite its favorable efficacy, clinical use is often associated with post-remission cytopenia, frequently necessitating treatment delays and dose modifications. This study aims to evaluate the efficacy and safety of shortened venetoclax treatment durations. A multicenter analysis was conducted involving 20 adult AML patients receiving venetoclax (7 or 14 days with 9 and 11 patients, respectively) combined with 5-azacitidine (5–7 days) between 2021 and 2024. The cohort included patients from four German academic centers all treated in first line. Outcome measures included bone marrow response, transfusion dependence, overall survival (OS) and progression-free survival (PFS). Median age was 73.5 years, with 70% of patients having secondary AML. Adverse molecular risk was observed in 75% of patients. The overall response rate (ORR) was 100%, with a composite complete remission rate of 78%. No significant differences in response rates were observed between the 7-day and 14-day venetoclax regimens. Median OS for the cohort was 15 months. Infection-related complications were observed in 55% of patients, with severe sepsis in 20% of cases. In this cohort, shortened venetoclax regimens demonstrated efficacy comparable to standard treatment protocols, with a potential reduction in hematologic toxicity. These findings support the individualization of treatment regimens to optimize clinical outcomes while potentially minimizing adverse effects.

**Supplementary Information:**

The online version contains supplementary material available at 10.1007/s00277-024-06048-5.

## Introduction

The combination of venetoclax (VEN) and hypomethylating agents (HMA) as 5-azacitidine (AZA) remains the standard of care for elderly patients with acute myeloid leukemia (AML) ineligible for intensive chemotherapy [1]. In the VIALE-A trial, the majority of patients (87%) exhibited post-remission cytopenia. Consequently, 77% experienced delays in subsequent treatment cycles, underscoring clinically relevant adverse hematologic effects of this combination regimen and the necessity for dosing adjustments, especially in responding patients [2].

Recently, numerous retrospective analyses have documented increasing clinical experience with this combination therapy. The substantial rate of treatment discontinuation due to venetoclax-related hematologic toxicities underscores the challenges inherent in this therapeutic approach [3]. A significant proportion of patients require VEN dose reductions or discontinue therapy due to cytopenia, especially neutropenia leading to infectious complications [4, 5].

Furthermore, even when complete remission (CR) or complete remission with incomplete blood count recovery (CRi) is achieved, red blood cell or platelet transfusion independence is rare (transfusion dependency of 70% for red blood cells and 58.6% for platelets under treatment). Prolonged hypoplastic bone marrow suppression during treatment is common, attributable to the significant toxicity of VEN/AZA on normal hematopoiesis. During treatment, a significant proportion (71.8%) of patients remain neutropenic for more than 30 days, frequently resulting in grade > 2 infectious complications (59.1%) [6].

Given the promising efficacy of novel FLT3 or IDH1/2 inhibitors, triplet combinations of targeted therapies with VEN/AZA have shown excellent clinical outcomes. However, these combinations have also been associated with prolonged absolute neutrophil recovery times, extending up to several weeks to months, resulting in a high risk of infectious complications [7, 8]. Therefore, shorter-duration protocols for VEN should be implemented to minimize toxicity in these settings.

For elderly and/or frail patients with limited bone marrow reserve, reduced-intensity strategies are particularly warranted. A once-weekly addition of venetoclax to decitabine has shown an overall response rate (ORR) of 88% for first-line treatment in AML patients. Compared to standard VEN/AZA dosing, a trend towards longer treatment duration and higher rates of transfusion independence has been shown [9]. Furthermore, current trials evaluating venetoclax in myelodysplastic neoplasm (MDS) are based on protocols using 14-days courses, due to the observed high rates of infectious complications observed in MDS patients [10]. Such metronomic dosing of VEN in combination with HMA treatment may potentially lead to entirely different pharmacokinetic and biological mechanisms, which require further detailed investigation.

In this multicenter analysis, we sought to evaluate the efficacy of shorter duration of venetoclax in first-line AML.

## Patients and methods

### Patient cohort

**Fig. 1:**
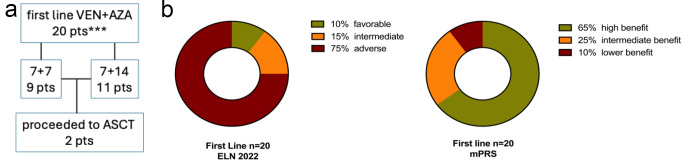
**a** Treatment diagram. Overview about different treatment cohorts and disease settings: * 2 patients without bone marrow assessment. VEN venetoclax; AZA 5-azacytidine; pts patients; ASCT allogeneic stem cell transplantation, 7 + 7 seven days venetoclax + seven days HMA; 7 + 14 seven days HMA + 14 days venetoclax.**b** Risk stratification. *ELN* European Leukemia Net; *mPRS* molecular prognostic risk score

Twenty adult AML patients receiving first-line treatment with VEN for either 7 or 14 days in addition to AZA for 5–7 days from 2021 to 2024 at four german academic sites were retrospectively assessed (Fig. 1a). Treatment decisions were based on the discretion of the physician, considering patient fitness and comorbidities (e.g., reduced renal or heart function), AML subtype (de novo vs. secondary AML/myeloproliferative neoplasia (s-AML/MPN)) in blast phase) or prior treatment with subsequent severe cytopenia. Patients excluded from the study where those not initially intended for a shortened VEN regimen upfront, but whose treatment was interrupted after 7 up to 14 days due to complications, as well as those lacking essential response and follow-up data. Additionally, patients who were switched to VEN/AZA regimen only after achieving complete remission and patients receiving VEN/AZA following VEN-containing induction treatment were excluded.

### Informed consent

All patients provided written informed consent for data acquisition and analysis. They were all included in the SAL (Study Alliance Leukemia) registry. The analysis was approved by the local ethics committee of Jena University Hospital, Germany (no. 3967–2/13 and 2024–3235) and of Würzburg University Hospital, Germany (no. 20240206 01).

### Patient treatment

VEN was administered orally for either 7 or 14 days during the first treatment cycle, in combination with AZA for 5 or 7 days (75 mg/m² subcutaneously), repeated in 28-day cycles.

### Cytogenetic and molecular genetic analysis

Cytogenetic evaluation was performed using standard Giemsa banding techniques, and karyotypes were described according to the current International System for Human Cytogenetic Nomenclature [11]. Detection of AML-associated molecular aberrations was performed by next-generation sequencing (NGS) according to European LeukemiaNet (ELN) 2022 guidelines [12]. Genetic characteristics prior to initiating VEN/AZA treatment are reported in Table 1.

### Response assessment

Response assessment by bone marrow analysis was primarily based on morphologic determination of blast count. Efficacy assessments included overall survival (OS), progression-free survival (PFS), and survival from the start of VEN treatment to the last follow-up or death from any cause. The overall response rate (ORR) was defined as CRc and partial remission (PR). PFS was the period from the initiation of VEN to progression/relapse or last follow-up/death from any cause [12]. Subgroup analyses were conducted based on the status of s-AML and the ELN 2022 risk group. In addition to ELN recommendations the recently developed molecular prognostic risk score (mPRS) for non-intensively treated patients was also applied for risk stratification. The score is based on three prognostic risk signatures associated with the mutational status of the four genes: TP53, NRAS, KRAS, and FLT3-ITD, indicating a lower, intermediate, or higher benefit from the treatment [13, 14].

CR was defined as ≤ 5% blasts in the bone marrow and adequate peripheral blood counts (neutrophils ≥ 1.0 × 10^9^/L, platelets ≥ 100 × 10^9^/L). PR was defined as 5–25% blasts in the bone marrow and a total reduction of at least 50% of AML blasts. Progressive disease (PD) was defined as an increase in bone marrow and/or peripheral blast count or new extramedullary manifestations. Additionally, transfusion requirements for platelets and red blood cells (RBC) were assessed. Transfusion dependence was determined by the Gale criteria, defined as the need for ≥ 2 units per month over the prior 3 months [15].

### Statistics

Time-to-event analyses (OS, PFS) were estimated using the Kaplan–Meier method and compared using the log-rank test. Chi-square test was employed to assess the differences between categorical variables across the different groups. P values < 0.05 were considered statistically significant. Statistical analyses were performed using GraphPad Prism 8.0.2 (GraphPad Inc., San Diego, CA, USA).

## Results

### Baseline characteristics

Median age was 73.5 (range, 56–86) years for the patient cohort. ECOG performance status was 0–1 in 20% (4/20) and 2–4 in 80% (16/20) of patients. At baseline, 30% (16/20) of patients had de novo AML, while the majority (70%, 14/20) had s-AML derived from MDS or blast phase MPN. Additionally, 25% (5/20) had AML post cytotoxic therapy (AML-pCT). Baseline characteristics are shown in Table 1.

**Table 1:** Patients characteristics

n	20	%
**Female**	**9**	45
**Median age**, **years (range)**	**73.5**	(56–86)
**ECOG**		
0–1	**4**	20
2–4	**16**	80
** s-AML**	**14**	70
**AML-pCT**	**5**	25
**7 + 7**	**9**	45
**7 + 14 (AZA + VEN)**	**11**	55
**Median bone marrow blasts**	**80**	(13–95)
**ELN 2022**		
Favorable	**2**	10
Intermediate	**3**	15
Adverse	**15**	75
**mPRS**		
High benefit	**13**	65
Intermediate benefit	**5**	25
Lower benefit	**2**	10
**Genetics**		
*FLT3-ITD*	**4**	20
*NPM1*	**5**	25
*IDH1/2*	**3**	15
*TP53*	**2**	10
*ASXL1*	**8**	40
*RUNX1*	**4**	20
**Myelodysplasia related changes**	**11**	55
Other		
Complex aberrant karyotype	**2**	10
Normal karyotype	**11**	55
**Previous HMA treatment**	**3**	15
**Previous intensiv chemotherapy**	**0**	0
**ASCT**	**2**	10
*Conditioning regimen*		
RIC	**2**	10
*Remission prior ASCT*		
CR1	**2**	10
CR2		
SD		
**Remission prior start VEN**		
PD/BP	**20**	100
CR, MRD positive	0	0

*ECOG* Eastern cooperative oncology group; *AML-pCT* acute myeloid leukemia post cytostatic treatment; *s-AML* secondary acute myeloid leukemia; *VEN* venetoclax; *HMA* hypomethylating agents; *ASCT* allogeneic stem cell transplantation; *CR* complete remission; *SD* stable disease; *RIC* reduced intensity conditioning; *PD* progressive disease; *BP* blast persistence; *MRD* measurable residual disease; *mPRS* molecular prognostic risk score

According to ELN 2022 criteria, 10% (2/20) of the patients had favorable, 15% (3/20) had intermediate, and 75% (15/20) had adverse risk (Fig. 1b). Noteworthy, mPRS risk stratification indicates that a majority of 65% of the patients are within the high-benefit group, whereas 25% and 10% of patients exhibit intermediate and low benefit, respectively. *FLT3*-ITD, *NPM1*, and *IDH1/2* were the most mutated genes, while also high proportion of *ASXL1* and *RUNX1* mutations could be detected, indicating the high proportion of myelodysplasia-related AML (Table 1). In the s-AML cohort, adverse risk genetic aberrations were identified in the majority of patients (79%, 11/14) whereas for de novo AML 50% (3/6) of patients showed an adverse risk profile. Complex karyotypes were observed in 10% (2/20) of the patients, while a normal karyotype was present in 55% (11/20) of the total cohort [12].

Regarding previous treatments of the cohort, 15% (3/20) of patients had prior HMA exposure for MDS treatment.

The two patients who underwent ASCT after first-line treatment with VEN/AZA were transplanted in first CR and received reduced-toxicity conditioning (RIC) with treosulfan and fludarabine, with or without anti-thymocyte globulin (ATG) [16, 17].

### Treatment characteristics

The median follow-up time since AML diagnosis was 7 months (range 1–36) overall. Median time to first bone marrow response assessment was 29.5 days (range 17–253) while the median time to best response was 37 days (range 22–253). Relapse under VEN/AZA treatment occurred in 25% (5/20) of patients after a median time to relapse of 12 months (range 1–35). Dose adjustment of VEN was required in 60% of patients due to concomitant azole prophylaxis [18]. The maximum VEN dose (400 mg or 200 mg/100 mg with concomitant prophylaxis with isavuconazole or posaconazole, respectively) was applied.

Early death rates were 5% (1/20) within 4 weeks and 20% (4/20) within 8 weeks. The median treatment duration was 127 days (range 26–365) and the median number of treatment cycles was 3 (range 1–9). Subsequent treatments included gilteritinib in 2 patients, low-dose cytarabine in 2 patients, and HMA monotherapy in 1 patient (Table 2).

**Table 2:** Treatment results

n	20	%
**Time to first response**, **days (median**, **range)**	**29.5**	(17–253)
**Time to best response**, **days (median**, **range)**	**37**	(22–253)
**Relapse under treatment**	**5**	25
Time to relapse, months (median, range)	**12**	
**Non-relapse mortality**	**3**	15
**Follow up from start treatment**, **months (median**, **range)**	**6.5**	(1–35)
**Follow up since diagnosis AML**, **months (median**, **range)**	**7**	(1–36)
**Early death rate**		
4 weeks	**1**	5
8 weeks	**4**	20
**Treatment duration**, **days (median**, **range)**	**127**	(26–365)
**Treatment cycles (median**, **range)**	**3**	(1–9)
**Concomitant azole treatment**	**12**	60
**Treatment post VEN/AZA**		
ASCT	**0**	0
Salvage chemotherapy	**0**	0
Gilteritinib	**2**	10
LODAC	**2**	10
HMA mono	**1**	5
**Infectious complications**		
Infections	**11**	55
Fever in neutropenia	**11**	55
Pneumonia	**6**	30
Possible IFD	**2**	10
Probable IFD	**1**	5
Covid19	**2**	10
Sepsis	4*	20
*all leading to death		

*HMA* hypomethylating agent; *AZA* 5-azacytidine; *VEN* Venetoclax; *ASCT* allogeneic stem cell transplantation; *LODAC* low dose cytarabine; *IFD* invasive fungal disease

### Safety

Non-relapse mortality was 15% (3/20) for the total cohort with two patients died on sepsis in CR and one patient on COVID-19. Infections were observed in 55% (11/20) of patients. Fever in neutropenia occurred in 55% (11/20) of patients, while pneumonia was reported in 30% (6/20). Possible invasive fungal disease (IFD) was noted in 10% (2/20) of patients, with probable IFD in 5% (1/20). Symptomatic COVID-19 infection were documented in 10% (2/20) of patients. Severe sepsis was recorded in 20% (4/20) of the patients, all leading to death.

### Response assessment

**Fig. 2:**
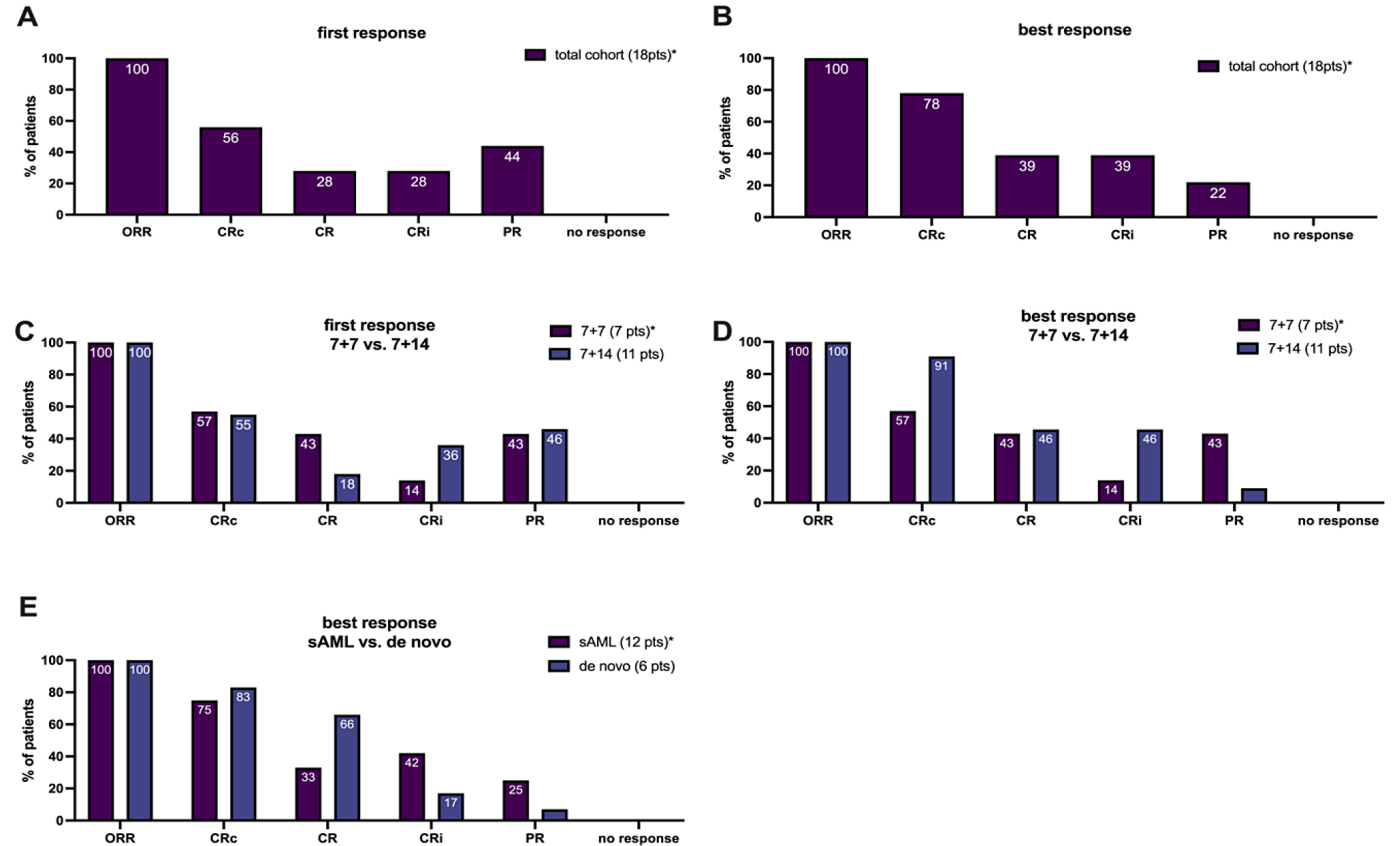
Response assessment. Bone marrow response rates for indicated patient cohorts at first and best response **A**-**E** are illustrated. *2 patients are excluded due to lack of bone marrow response data. *ORR* Overall response rate; *CRc* composite complete remission (CR + CRi); *CR* complete remission; *CRi* complete remission with incomplete hematological recovery; *PR* partial remission; *sAML* secondary acute myeloid leukemia

Bone marrow response both at first and best response assessments showed an ORR rate of 100% (18/18, Fig. 2, A and B). The CRc rate improved from of 56% (10/18) at first assessment to 78% (14/18) at the time of best response. Of note, 22% (4/18) of patients who had a PR at the first assessment achieved a CR or CRi at the time of best response in this group. Regarding first and best response assessment, no differences between non-responders could be documented.

Patients with s-AML were more likely to achieve a CRi (42%, 5/12) than CR (33%, 4/12) while 66% (4/6) of de novo AML achieved CR and only 17% (1/6) CRi. The CRc rate was 83% (5/6) for de novo AML and 75% (9/12) for s-AML (Fig. 2E).

### Comparison of 7 + 7 vs. 7 + 14

**Fig. 3:**
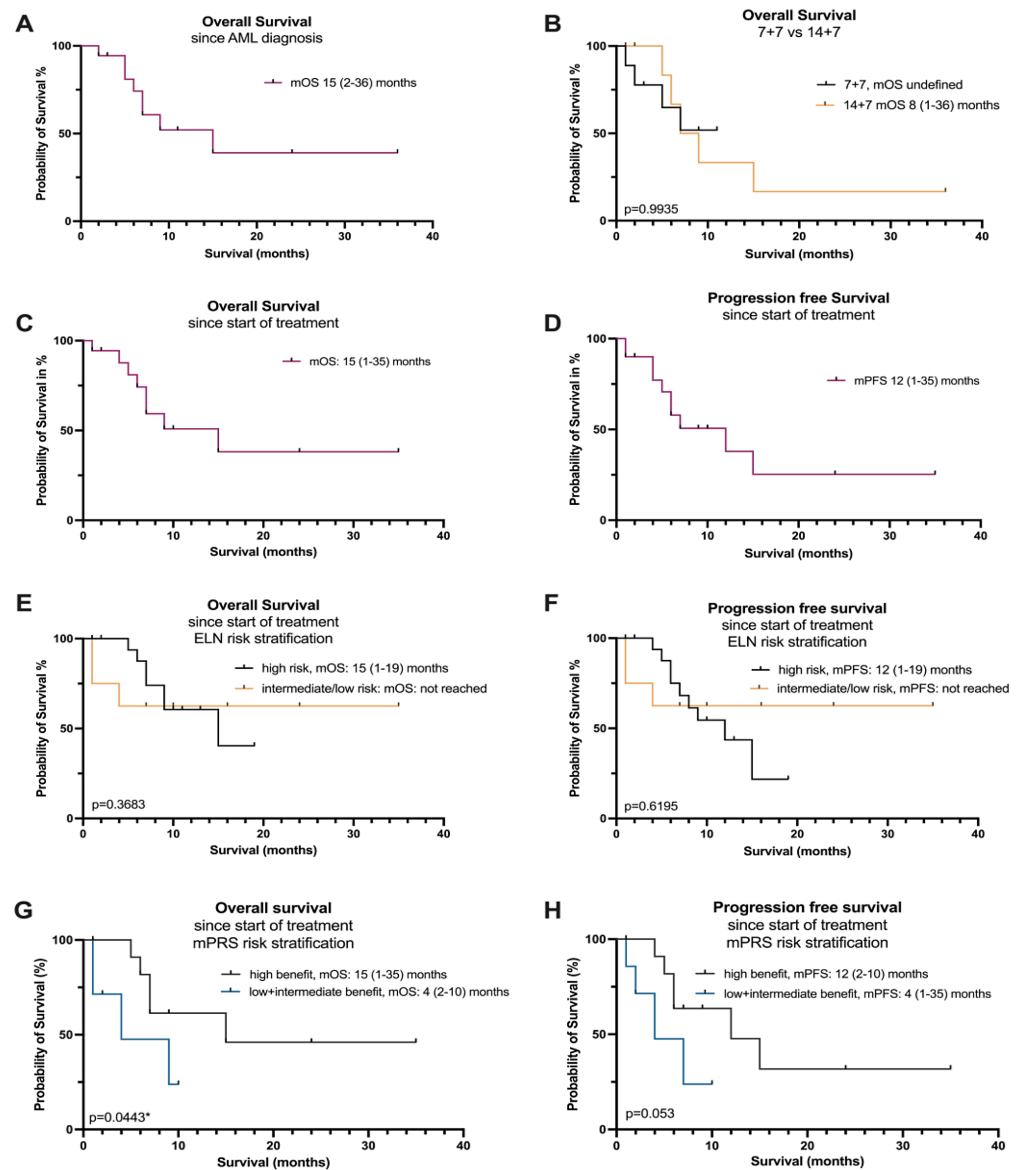
Survival analysis. Kaplan-Meier estimates for OS **A**-**C** and PFS **D** are illustrated for indicated treatment cohorts. In **E** and **F**, OS and PFS for ELN2022 and in **G** and **H** mPRS risk groups are shown.*mOS* median overall survival; *mPFS* median progression free survival, *ELN* European leukemia Net *mPRS* molecular prognostic risk score

When comparing response rates based on VEN exposure duration (7 vs. 14 days) no significant differences in CRc rate were found (*p* = 0.267) (Fig. 2, C and D). The differences regarding CRc rate at the time of the first response was 57% vs. 55% and 57% vs. 91% for best response for 7 + 7 and 14 + 7, respectively; *p* = 0.752). No significant differences were seen when comparing the OS for 7 + 7 and 14 + 7 patients of the first-line group (*p* = 0.99), (Fig. 3B).

### Survival analysis

Median OS from start of VEN/AZA treatment was 15 months (range 1–35) for the treatment cohort (Fig. 3A, C) and the median PFS was 12 months (range 1–35) (Fig. 3D). Comparing the survival according to ELN 2022 risk groups, OS and PFS was 15 (range 1–19) and 12 months (range 1–19) for the adverse risk patients, respectively, and not reached for the intermediate and low risk cohort (Fig. 3, E and F, *p* = 0.37 and *p* = 0.62, respectively). Calculation for the mPRS risk stratification demonstrates a statistically significant longer OS (*p* = 0.044) and a trend for PFS (*p* = 0.053) for the high benefit group compared to intermediate and low benefit patients (Fig. 3G, H).

### Impact on blood count and transfusion dependency

Transfusion dependency for platelets and RBC as well as neutrophil counts were monitored (Table 3). Prior to treatment 25% (5/20) of patients required substitution for platelets with 15% (3/20) by day 30. For RBC, 55% (11/20) of patients required transfusions prior to VEN/AZA initiation, reducing to 45% (9/20) by day 30 and 15% (3/12) by day 90. The median for platelets prior to treatment was 69 × 10^9^/L and 207.5 × 10^9^/L at day 30, while the median for RBC was increasing from 5.6 × 10^9^/L to 5.95 × 10^9^/l on day 60. Neutrophil counts increased from a median of 0.45 × 10^9^/L on day 0 to 1.43 × 10^9^/L by day 90.

Of patients achieving CRc, among eight evaluable patients, 75% and 62% did experience grade 3 and 4 thrombocytopenia, respectively, and 75% did experience grade 4 neutropenia. Hematopoietic recovery in these patients was achieved with platelet counts exceeding 50 × 10⁹/L after a median of 27 days (range 14–45 days), and neutrophil counts exceeding 1 × 10⁹/L after a median of 29 days (range 4–46 days). After a median time of 31.5 days (range 32–53 days) those patients continued with the subsequent VEN/AZA course.

**Table 3:** Blood count response

	plt d0 (20 pts)	plt d30 (20 pts)	plt d60 (12 pts)	plt d90 (12pts)
substitution, n (%)	5 (25)	3 (15)	3 (25)	2 (16)
Platelets [10^9^/l], median (range)	69 (27–218)	207 (31–488)	170 (50–348)	181 (24-1130)
	**RBC d0**	**RBC d30**	**RBC d60**	**RBC d90**
substitution, n (%)	11 (55)	9 (45)	6 (50)	3 (25)
Hemoglobin [mmol/l] median (range)	5.6 (4.7–7.4)	5.7 (4.8–7.6)	5.95 (5.4–8.3)	6.3 (4.7–7.5)
	**neutrophils d0**	**neutrophils d30**	**neutrophils d60**	**neutrophils d90**
Neutrophils [10^9^/l] median (range)	0.45 (0.1–15)	0.76 (0.01-35)	1.5 (0.1–9.6)	1.43 (0.1–4.7)

*Plt* platelets; *pts* patients; *RBC* red blood cells

## Discussion

This multicenter analysis provides critical insights into efficacy and safety of shorter-duration in combination with HMA in first-line AML. Our findings align with existing literature, indicating that reduced-duration VEN regimens can achieve substantial responses while potentially reducing hematological toxicity associated with longer VEN exposure [19–23]. The CRc rate of 78% is comparable to previously reported outcomes with standard and reduced VEN/HMA protocols. A recent study on 36 newly diagnosed AML patients from China showed a CRc rate of 66.7% with 100 mg VEN for 14 days in combination with standard dose azacitidine [21]. Another report indicated a CRc rate of 76.9%, with no differences when comparing 14-days vs. 28-days VEN exposure [22]. The most recent retrospective data compared 82 patients who received first-line treatment with a ‘7 + 7 regimen’ to a cohort of 173 patients treated with the standard dose VEN/HMA. While no differences in response rates were observed between the groups, the ‘7 + 7 regimen’ was associated with significantly lower platelet transfusion requirements and a reduced 8-week mortality rate [24, 25]. Notably, our study revealed no significant differences in ORR and CRc between patients receiving 7-days vs. 14-days VEN courses, suggesting that shorter-duration VEN is equally effective.

In patients with s-AML, the CR rate was notably lower than in those with de novo AML, highlighting the inherent challenges in treating s-AML. This discrepancy may be attributed to the more adverse genetic profiles observed in the s-AML group, consistent with previous studies indicating poorer outcomes in this subgroup [26–28]. Despite this, the high overall CRc rate of 75% in 14-AML patients underscores the potential of VEN/HMA regimens even in genetically adverse-risk populations. Noteworthy, the novel mPRS risk stratification for non-intensively treated patients revealed a significant prolonged OS of the high-benefit group within our cohort. This classification contrasts markedly with the distribution of ELN risk groups, highlighting the potential clinical relevance of this risk model.

The median overall survival of 15 months in first-line patients not eligible for intensive treatment approaches in our study is comparable to the 14.7 months observed in the VIALE A trial. This finding is of particularly noteworthy given the higher proportion of genetically adverse-risk patients, including those with s-AML, in our study. Specifically, 70% of patients had s-AML compared to 25% in the VIALE A trial. Our cohort primarily consists of highly comorbid patients, with 80% (16/20) having an ECOG score of 2–4, including those with significant organ function impairment which led physicians to shorten the duration of VEN treatment. Our data suggest that reduced VEN courses could be particularly beneficial for patients who would not meet the inclusion criteria for clinical trials.

As expected, improved neutrophil count and reduced transfusion dependency were noted over time. A more detailed analysis of a subset of patients who achieved CRc revealed a median time of 31.5 days before continuing the next course of VEN/AZA, compared to 34 days in the VIALA-A trial. Compared to our previous analysis investigating standard dose VEN, shorter-duration VEN regimens appear to support quicker hematologic recovery [29]. Notably the median platelet count normalized (181 × 10^9^/l (24–1130) after 3 cycles of shorter-duration VEN compared to 42.5 × 10^9^/l (12–280) with standard VEN dosage. Additionally, the median neutrophil count 3 months after initiation of VEN/HMA treatment was in the reduced regimen 1.43 × 10^9^/l (0.1–4.7) versus 0.2 × 10^9^/l (0.1–3.8) in standard VEN regimen. Comparing the homogeneous first-line cohorts of the shortened-duration VEN/AZA regimen to the standard dose shows higher bone marrow response rates and improved OS and PFS in the shortened duration VEN group, even with comparable risk profiles [29]. The increased clinical experience with VEN/AZA treatment may have contributed to the improved outcomes documented in the more recent ‘VEN reduced’ cohort (Table S1).

The feasibility of reduced VEN protocols has also been demonstrated as maintenance therapy in AML patients not immediately eligible for ASCT [30]. In a phase 2 trial, 35 patients who achieved a CRc following intensive or low-intensity treatment received either 7 or 14 days of VEN in combination with azacitidine (50mg/m^2^). The 2-year relapse-free survival was 65% (95% CI 50–85). Future studies should focus on optimizing the balance between efficacy and toxicity by exploring various VEN dosing schedules and combinations with other novel agents such as combinations with FLT3, IDH1/2, or Menin inhibitors [31–34].

Regarding infectious complications, a descriptive comparison with a retrospective analysis of 118 palliative AML patients at our institution was conducted. This cohort consisted mostly of patients treated with HMA monotherapy (85%) [35]. In this group, the incidence of general infections was observed to be 60.2%, with pneumonia occurring in 37.3% of cases. Possible and probable IFD were identified in 11% and 0.8% of cases, respectively, while 4.2% experienced sepsis. These results are comparable to those of our currently presented cohort, with comparable rates for general infections and pneumonia (55% and 30%), but slightly higher rates for possible and probable IFD (10% and 5%) and sepsis (20%). This comparison suggests that reduced VEN/HMA protocols do not result in significantly higher infectious complications compared to HMA monotherapy.

Our study provides a comparable follow-up time period of 7 months, in line with similar published analyses reporting follow-up durations of 4.7 and 7.7 months, underscoring the need for longer-term observations to fully assess the durability of responses and long-term safety of reduced-duration VEN protocols [19, 22]. Continued monitoring and reporting of registry data will be crucial in refining treatment strategies and improving outcomes for this vulnerable patient population.

In conclusion, this study contributes to the growing body of evidence supporting the efficacy and safety of shortened-duration VEN in combination with AZA for AML treatment. These findings advocate for individualized treatment approaches that consider patient fitness, genetic risk profiles, and potential for hematologic recovery, ultimately aiming to optimize clinical outcomes while minimizing adverse effects.

## Electronic supplementary material

Below is the link to the electronic supplementary material.

Supplementary Material 1

## Data Availability

No datasets were generated or analysed during the current study.
