# Correction to: Azacitidine in combination with shortened venetoclax treatment cycles in patients with acute myeloid leukemia

**DOI:** 10.1007/s00277-024-06095-y

**Published:** 2024-11-20

**Authors:** Maximilian Fleischmann, Madlen Jentzsch, Annamaria Brioli, Florian Eisele, Jochen J Frietsch, Farina Eigendorff, Romy Tober, Karin G Schrenk, Jakob Friedrich Hammersen, Olaposi Yomade, Inken Hilgendorf, Andreas Hochhaus, Sebastian Scholl, Ulf Schnetzke

**Affiliations:** 1 Klinik für Innere Medizin II, Abteilung für Hämatologie und Internistische Onkologie, Universitätsklinikum Jena, Jena, Germany; 2 Comprehensive Cancer Center Central Germany, Campus Jena, Jena, Germany; 3 Klinik und Poliklinik für Hämatologie, Zelltherapie, Hämostaseologie und Infektiologie, Universitätsklinikum Leipzig, Zelltherapie, Leipzig, Germany; 4 Comprehensive Cancer Center Central Germany, Campus Leipzig, Leipzig, Germany; 5 Klinik und Poliklinik für Innere Medizin C, Abteilung für Hämatologie und Onkologie, Universitätsklinikum Greifswald, Greifswald, Germany; 6 Medizinische Klinik und Poliklinik II, Universitätsklinikum Würzburg, Würzburg, Germany

**Correction to: Annals of Hematology**

10.1007/s00277-024-06048-5

The affiliations of the first two authors were not correctly represented. Please find below the correct author affiliation details for reference. We kindly request an update to reflect these corrections in the article.

Maximilian Fleischmann^1,2^; Madlen Jentzsch^3,4^; Annamaria Brioli^5^; Florian Eisele^6^; Jochen J Frietsch^6^; Farina Eigendorff^1,2^; Romy Tober^1,2^; Karin G Schrenk^1,2^; Jakob Friedrich Hammersen^1,2^; Olaposi Yomade^1,2^; Inken Hilgendorf^1,2^; Andreas Hochhaus^1,2^; Sebastian Scholl^1,2^; Ulf Schnetzke^1,2^.

^1^ Klinik für Innere Medizin II, Abteilung für Hämatologie und Internistische Onkologie, Universitätsklinikum Jena, Jena, Germany.

^2^ Comprehensive Cancer Center Central Germany, Campus Jena, Jena, Germany.

^3^ Klinik und Poliklinik für Hämatologie, Zelltherapie, Hämostaseologie und Infektiologie, Universitätsklinikum Leipzig, Leipzig, Germany.

^4^ Comprehensive Cancer Center Central Germany, Campus Leipzig, Leipzig, Germany.

^5^ Klinik und Poliklinik für Innere Medizin C, Abteilung für Hämatologie und Onkologie, Universitätsklinikum Greifswald, Greifswald, Germany.

^6^ Medizinische Klinik und Poliklinik II, Universitätsklinikum Würzburg, Würzburg, Germany.

The original article has been corrected.

