# Supplementary material for: Azacitidine in combination with shortened venetoclax treatment cycles in patients with acute myeloid leukemia
Source: Ann Hematol. 2024 Oct 25;104(1):285–94. doi: 10.1007/s00277-024-06048-5 (PMC11868173; doi:10.1007/s00277-024-06048-5)
Supplement: Supplementary file 1 — Supplementary Material 1 [file 277_2024_6048_MOESM1_ESM.docx]

**Table S1** Data comparison of reduced and standard dose regimen as first line treatment

|  | **7+7/7+14 (n=20)** | **Standard dose 28d (n=17)** |
| --- | --- | --- |
| **Median Age , years (range)** | 73.5 (56-86) | 77 (54-83) |
| **Number of treatment cycles, median (range)** | 3 (1-9) | 3 (1-15) |
| **mPRS in %** |  |  |
| High benefit | 65 | 76 |
| Intermediate benefit | 25 | 18 |
| Lower benefit | 10 | 6 |
| **Follow up from start treatment, months (range)** | 6.5 (1-35) | 3.26 (2-21) |
| **Early death rate at 4 weeks, (%)** | 1 (5) | 0 (0) |
| **Bone marrow response** |  |  |
| ORR | 18 (100) | 10 (59) |
| CRc | 14 (78) | 5 (30) |
| CR | 7 (39) | 3 (18) |
| Cri | 7 (39) | 2 (17) |
| PR | 4 (22) | 5 (30) |
| No response | 0 | 6 (35) |
| **OS** | 15 (2-36) | 13.3 (2.2-20.5) |
| **PFS** | 12 (1-35) | 5.7 (1.4-20.5) |

*mPRS* molecular prognostic risk score, *ORR* Overall response rate; *CRc* composite complete remission (CR+CRi); *CR* complete remission; *CRi* complete remission with incomplete hematological recovery; *PR* partial remission;
